# LUMiC Endoprosthetic Reconstruction of Periacetabular Tumor Defects: A Multicenter Follow-up Study

**DOI:** 10.2106/JBJS.23.01082

**Published:** 2024-05-23

**Authors:** Richard E. Evenhuis, Michiel A.J. van de Sande, Marta Fiocco, Edwin F. Dierselhuis, Demien Broekhuis, Michaël P.A. Bus

**Affiliations:** 1 Department of Orthopaedic Surgery, Leiden University Medical Center, Leiden, The Netherlands; 2 Center for Pediatric Oncology, Prinses Maxima Center, Utrecht, The Netherlands; 3 Mathematical Institute, Leiden University, Leiden, The Netherlands; 4 Medical Statistics, Department of Biomedical Science, Leiden University Medical Center, Leiden, The Netherlands; 5 Department of Orthopaedic Surgery, Radboudumc, Nijmegen, The Netherlands

## Abstract

**Update:**

This article was updated on July 17, 2024 because of a previous error, which was discovered after the preliminary version of the article was posted online. The byline that had read “Richard E. Evenhuis, MD^1^, Michiel A.J. van de Sande, MD, PhD^1,2^, Marta Fiocco, PhD^2,3,4^, Demien Broekhuis, MD^1^, Michaël P.A. Bus, MD, PhD^1^, and the LUMiC® Study Group*” now reads “Richard E. Evenhuis, MD^1^, Michiel A.J. van de Sande, MD, PhD^1,2^, Marta Fiocco, PhD^2,3,4^, Edwin F. Dierselhuis, MD, PhD^5^, Demien Broekhuis, MD^1^, Michaël P.A. Bus, MD, PhD^1^, and the LUMiC® Study Group*”. The Department of Orthopaedic Surgery, Radboudumc, Nijmegen, The Netherlands, has been added as the affiliation for Edwin F. Dierselhuis, MD, PhD.

**Background:**

We previously reported promising early results for periacetabular tumor reconstructions using the LUMiC prosthesis. The current study evaluates mid-term complications, revision rates, cumulative incidence of implant revision, and risk factors for complications in a multicenter cohort.

**Methods:**

We assessed patients in whom a tumor defect after type P1b+2, P2, P2+3, or P1b+2+3 internal hemipelvectomy was reconstructed with a LUMiC prosthesis during the period of 2008 to 2022. Complications were reported according to the Henderson classification. Competing risks models were used to estimate the cumulative incidence of implant revision for mechanical and nonmechanical reasons, and reoperations for any complication. Cox models were used to study the effect of risk factors on dislocation and infection.

**Results:**

One hundred and sixty-six patients (median follow-up, 4.2 years [interquartile range, 2.6 to 7.6 years]) were included. A total of 114 (69%) were treated for a primary malignant tumor, 46 (28%) for metastatic carcinoma, 5 (3%) for a benign aggressive lesion, and 1 (1%) for another reason. One hundred and sixty-five reoperations were performed in 82 (49%) of the patients; 104 (63%) of the reoperations were within 6 months. Thirty-two (19%) of 166 implants were revised: 13 (8%) for mechanical reasons, mainly dislocation (n = 5, 3%), and 19 (11%) for nonmechanical reasons, mainly periprosthetic joint infection (PJI) (n = 15, 9%). The cumulative incidences of revision for mechanical reasons and PJI (Henderson 1 to 4) at 2, 5, and 10 years were 11% (95% confidence interval [CI], 7% to 17%), 18% (12% to 25%), and 24% (16% to 33%), respectively. Previous surgery at the same site was associated with an increased dislocation risk (cause-specific hazard ratio [HR_CS_], 3.0 [95% CI, 1.5 to 6.4]; p < 0.01), and resections involving the P3 region were associated with an increased infection risk (HR_CS_, 2.5 [95% CI, 1.4 to 4.7]; p < 0.01).

**Conclusions:**

Despite a substantial reoperation risk, the LUMiC prosthesis demonstrated its durability in the mid-term, with a low mechanical revision rate and most patients retaining their primary implant. Most complications occur in the first postoperative months. Patients with previous surgery at the same site had an increased dislocation risk and might benefit from more conservative rehabilitation and aftercare. Measures should be aimed at reducing the PJI risk, especially in resections involving the P3 region.

**Level of Evidence:**

Therapeutic Level IV. See Instructions for Authors for a complete description of levels of evidence.

Over the past decades, nonmodular stemmed acetabular cups have gained popularity for the reconstruction of periacetabular tumor defects because of their wide availability, intraoperative flexibility, relatively fast and easy implantation, and the possibility of allowing early weight-bearing and rapid postoperative mobilization^1,2^. Nevertheless, as with any periacetabular reconstruction technique, the risks of dislocation (3% to 31%), aseptic loosening (0% to 16%), and periprosthetic joint infection (PJI) (10% to 50%) remain substantial. These complications commonly necessitate revision surgery, resulting in an even higher risk of complications and morbidity^1-11^.

The LUMiC prosthesis (Implantcast) was introduced in 2008 for the reconstruction of extensive periacetabular defects. This modular device consists of a stem that sits in the remaining ilium, in line with the weight-bearing axis of the pelvis, and an acetabular cup that is connected to the stem^12^. The stem and cup are equipped with a sawtooth junction allowing for rotational adjustment of the cup. In a previous study^2^, we reported promising short-term complication and implant survival rates compared with other techniques^1,4,13-15^.

In the current study, we aimed to assess the mid-term results of this implant, in a larger multicenter cohort. Therefore, we evaluated (1) the complications and associated risk factors, (2) the reasons for implant revision, and (3) the cumulative incidence of implant revision at 2, 5, and 10 years.

## Materials and Methods

Approval for conducting this study was obtained from the scientific committee of the Leiden University Medical Center (LUMC). The committee waived patients’ informed consent (W.22.002/2022-029). Participating centers obtained approval by their local ethical review board.

### Study Design, Setting, Participants

In this international, multicenter, observational retrospective study, we assessed all patients in whom an internal hemipelvectomy was performed for a bone tumor and in whom the LUMiC prosthesis was used for reconstruction of the defect during the period of 2008 to 2022. The minimum potential follow-up was 24 months; patients who died within 24 months after implantation were included. Fourteen tertiary referral centers participated. All patients had a periacetabular tumor defect (including P2 according to the modified Enneking classification^16^) in which the medial ilium was preserved as described in our previous work^2,4^. One hundred and sixty-six patients (87 female, 52%) with a median age of 57 years (range, 10 to 81 years) were included. The median follow-up for censored patients was 4.2 years (interquartile range [IQR], 2.6 to 7.6 years). The indication for reconstruction in 114 (69%) of the patients was resection of a primary malignant bone tumor, while 46 (28%) had been treated for metastatic carcinoma (Table I). Twenty-six (16%) of the patients had ≥1 previous surgeries at the same site (Table I). All patients received prophylactic antibiotics according to the local protocol: either as a single dose (n = 25, 15%) or over 24 hours (n = 55, 34%), 3 to 5 days (n = 53, 33%), or >5 days (n = 29, 18% [of 162 with data on antibiotic duration]) (Table II). According to the modified Enneking classification, 17 (11%) of the patients underwent type P1b+2 resection; 83 (52%), type P2 resection; 50 (31%), type P2+3 resection; and 11 (7%), type P1b+2+3 resection^4,16^. In 60 (41%) of patients (146 with data), an extra-articular resection of the hip joint was performed.

**TABLE I:** Study Population (N = 166)*

Variable	Values
Sex	166
Male	79 (48%)
Female	87 (52%)
BMI† *(kg/m*^*2*^*)*	25 [22-28]
ASA score	165
1	40 (24%)
2	81 (49%)
3	44 (27%)
Smoking	139
Yes, currently	20 (14%)
Yes, formerly (stopped >6 mo.)	17 (12%)
Diabetes	12/152 (8%)
Indication for reconstruction	166
Primary malignant tumors	114 (69%)
Chondrosarcoma	67 (40%)
Osteosarcoma	18 (11%)
Ewing sarcoma	11 (7%)
Soft-tissue sarcoma	9 (5%)
Multiple myeloma	4 (2%)
Other	5 (3%)
Metastatic carcinoma	46 (28%)
Benign aggressive lesions	5 (3%)
GCTB	2 (1%)
Chondroblastoma	1 (1%)
Chondromyxoid fibroma	1 (1%)
Yeast infection‡	1 (1%)
Other	1 (1%)
Previous surgery at same site	26 (16%)
Internal hemipelvectomy or partial pelvic tumor resection	9 (35%)
Total hip arthroplasty	13 (50%)
Curettage (GCTB/osteoblastoma)	4 (15%)
Soft-tissue involvement	91/158 (55%)
Pathological fracture at diagnosis	30/162 (19%)
Neoadjuvant chemotherapy	55/163 (34%)
Neoadjuvant radiation therapy	28/163 (17%)
Adjuvant chemotherapy	52/162 (32%)
Adjuvant radiation therapy	30/159 (19%)

* The values are given as the number, with the percentage in parentheses, except where otherwise noted. BMI = body mass index, ASA = American Society of Anesthesiologists physical status, and GCTB = giant cell tumor of bone.

† BMI values are given as the median, with the interquartile range (IQR) in square brackets.

‡ Suspicious lesion in a patient with known multiple myeloma. Histology identified no tumor localization but a yeast infection.

**TABLE II:** Prosthesis and Surgical Details

Variable	No. (%)*
Antibiotic administration	161
Cephalosporins	100 (62%)
Beta-lactam	13 (8%)
Cephalosporins + clindamycin	12 (8%)
Cephalosporins + metronidazole	8 (5%)
Glycopeptides	10 (6%)
Cephalosporins + aminoglycosides	6 (4%)
Glycopeptides + β-lactam	6 (4%)
Other	6 (4%)
Modified Enneking resection type	161
P1b+2	17 (11%)
P2	83 (52%)
P2+3	50 (31%)
P1b+2+3	11 (7%)
Use of computer-assisted surgery	49/166 (30%)
Concomitant proximal femoral reconstruction	56/165 (34%)
Silver-coated proximal femur	40/56 (71%)
Cemented LUMiC stem	30/165 (18%)
Cup size	163
50 mm	62 (38%)
54 mm	49 (30%)
60 mm	52 (32%)
Dual-mobility cup	107/164 (65%)
Silver-coated cup	36/166 (22%)
Cemented femoral component	62/164 (38%)
Use of Trevira tube	48/163 (29%)

* Total cohort, n = 166.

### Preoperative Planning, Surgical Details, Procedure

The general surgical and procedural details were previously described^12^. Although not available in all participating centers, the leading center prefers the use of intraoperative navigation to optimize stem placement. Press-fit fixation of an uncemented stem was preferred unless adequate press-fit fixation of the stem was not obtained, or if bone quality was inadequate. Conventional and dual-mobility cup articulations were available and were used at the surgeon’s discretion, although the dual-mobility cup was preferred on the basis of previous results^2^. Depending on the surgeon’s preferences, a Trevira attachment tube (Implantcast) was used to reattach soft tissues. Usually, early partial weight-bearing (with use of 2 crutches) was allowed under supervision of a physiotherapist. At 6 to 12 weeks, this was gradually increased to full weight-bearing. Combined flexion and external rotation was avoided. In some centers, orthoses were used.

Generally, patients with a suspected PJI underwent a DAIR (debridement, antibiotics, and implant retention) procedure, including intraoperative culturing and a thorough debridement, followed by at least 2 weeks of intravenous antibiotics. The standard antibiotic treatment regimen spanned a minimum of 12 weeks, depending on the isolated microorganism(s) and the susceptibility pattern. In some cases, eradication of the PJI was not achieved, resulting in chronic antibiotic suppression or a draining fistula, as described in our previous paper^11^.

### Variables

Patient records were reviewed to obtain demographics, surgical details, reconstruction details, complications, and functional outcomes at the last date of follow-up. Incision types were divided into 2 groups: a single iliofemoral (“question mark”) incision or a star-shaped incision. Pelvic resection types were divided into 2 groups: P1b+2 and P2 or P1b+2+3 and P2+3. Revision was defined as any surgical procedure in which (part of) the implant was removed or replaced. Complications and the reason for implant revision were categorized according to the Henderson classification^17^.

### Statistical Analysis

Competing risks models^18^ were used to estimate the cumulative incidences of implant revision and reoperations. A competing risks model with 3 competing events was used to estimate the cumulative incidences of mechanical failure and infection, with death and local recurrence as competing events. A second competing risks model with 2 competing events was employed to estimate the cumulative incidence of any complication, with death as a competing event.

Cause-specific Cox hazard regression models were estimated to study the effect of prognostic risk factors on time to dislocation and time to PJI. Cause-specific hazard ratios and 95% confidence intervals are reported. The proportion of complications was compared among different subgroups using chi-square analysis. Analyses of data were performed using SPSS version 25.0 (IBM) and RStudio version 4.2.1^19^. The R Studio package “cmprsk” was used to estimate the cumulative incidence of implant revision and reoperations. The level of significance was set at p < 0.05.

## Results

### Complications, Implant Revision, Risk Factors

During the study period, 82 (49%) of the patients underwent ≥1 reoperation (Table III). In total, 165 reoperations were performed, of which 104 (63%) were within 6 months.

**TABLE III:** Complication and Revision Rates, Time to Revision, and Reconstruction Status Among Revised Cases

Complication*	No. (%)		Time to Revision After Implantation *(mo)*	Reconstruction Status at Latest Follow-up (No.)
	Patients	Revisions		
Total	82/166 (49%)	32/166 (19%)	0-99	
H1A (dislocation)	31 (19%)	5 (3%)	0-45	Revision LUMiC (3), resection arthroplasty (1), hindquarter amputation (1)†
H1B (aseptic wound dehiscence)	7 (4%)	0 (0%)		
H2A (aseptic loosening <2 yr after implantation)	1 (1%)	1 (1%)	12	Resection arthroplasty (1)
H2B (aseptic loosening ≥2 yr after implantation)	4 (2%)	4 (2%)	53-77	Custom-made implant (4)
H3A (implant breakage or wear)	1 (1%)	1 (1%)	32	Revision LUMiC (1)
H3B (periprosthetic osseous fracture)	2 (1%)	2 (1%)	0, 9	Revision LUMiC (1), resection arthroplasty (1)
H4A (PJI <2 yr after implantation)	36 (22%)	11 (7%)	0-20	Revision LUMiC (2), resection arthroplasty (6), custom-made implant (1), hindquarter amputation (1), spacer (1)
H4B (PJI ≥2 yr after implantation)	5 (3%)	4 (2%)	35-65	Revision LUMiC (1), resection arthroplasty (2), rotationplasty (1)
H5A (soft-tissue progression of tumor)	2 (1%)	0 (0%)		
H5B (osseous progression of tumor)	13 (8%)	4 (2%)	0-99	Hindquarter amputation (3), resection arthroplasty (1)
Other	12 (7%)	0 (0%)		

* H = Henderson classification, and PJI = periprosthetic joint infection.

† One patient had revision LUMiC for dislocation but later underwent amputation due to osseous progression of tumor.

Dislocations (Henderson 1A) occurred in 31 (19%) of the patients; 21 (13%) had a single dislocation and underwent closed or open reduction, and 10 (6%) had recurrent dislocations. The first dislocation occurred within 1 month in 2 (6%) of the 31 patients, between 1 and 6 months in 4 (13%), and later in 5 (16%). Patients who had previous surgery at the same site had a higher dislocation risk than those without previous surgery at the same site (cause-specific hazard ratio [HR_CS_], 3.0 [95% confidence interval (CI), 1.5 to 6.4]; p < 0.01) (Table IV). Utilization of the dual-mobility cup (HR_CS_, 0.6 [95% CI, 0.3 to 1.2]; p = 0.17) or the Trevira tube (HR_CS_, 0.7 [95% CI, 0.3 to 1.6]; p = 0.38) was not significantly associated with dislocation. Dislocations occurred in 15 (26%) of 57 patients with conventional cups compared with 16 (15%) of 107 with dual-mobility cups (p = 0.08). Dislocations occurred in 7 (15%) of 48 patients with reconstruction with a Trevira tube versus 24 (21%) of 115 without (p = 0.35). Dislocations occurred in 3 (10%) of 29 patients with reconstruction with a Trevira tube and dual-mobility cup versus 11 (30%) of 37 who had neither (p = 0.06). Five implants (3% of patients) were revised for instability. Four conventional cups (2%) were exchanged for a dual-mobility cup, and 1 (1%) resection arthroplasty was performed because of recurrent instability and poor oncological prognosis.

**TABLE IV:** Univariate Cause-Specific Cox Proportional Hazards Regression Model for Prognostic Factors for the Occurrence of Dislocation and PJI*

Possible Risk Factors	Dislocation	P Value	PJI	P Value
	HR_CS_ (95% CI)		HR_CS_ (95% CI)	
Sex				
Female†				
Male	1.8 (0.89-3.77)	0.10	1.2 (0.66-2.21)	0.54
Age	1.0 (0.99-1.05)	0.07	1.0 (1.00-1.04)	0.05
BMI	1.0 (0.96-1.10)	0.49	1.0 (0.98-1.10)	0.21
ASA classification				
I†				
II	1.3 (0.56-3.20)	0.52	1.2 (0.57-2.73)	0.59
III	0.8 (0.28-2.51)	0.76	1.5 (0.62-3.49)	0.39
Smoking	NA			
No†				
Yes			0.5 (0.16-1.68)	0.27
Diabetes	NA			
No†				
Yes			1.9 (0.69-5.63)	0.20
Previous surgery at same site				
No†				
Yes	3.0 (1.47-6.41)	0.003	0.8 (0.34-1.89)	0.61
Incision type	NA			
Single incision†				
Star-shaped incision			1.3 (0.69-2.57)	0.39
Proximal femoral resection				
No†				
Yes	0.8 (0.35-1.64)	0.48	1.2 (0.66-2.26)	0.53
Type of pelvic resection				
P1b+2 and P2†				
P2+3 and P1b+2+3	1.4 (0.70-2.95)	0.32	2.5 (1.35-4.72)	0.004
Surgical duration *(hr)*	NA		1.1 (0.98-1.29)	0.09
Blood loss *(L)*	NA		1.1 (0.84-1.32)	0.67
Dual-mobility cup				
No†				
Yes	0.6 (0.29-1.23)	0.17	NA	
Use of silver-coated cup	NA			
No†				
Yes			2.1 (1.11-4.04)	0.02
Use of silver-coated proximal femur	NA			
No†				
Yes			0.2 (0.07-0.49)	<0.001
Use of computer-assisted surgery				
No†				
Yes	0.7 (0.38-1.92)	0.71	0.8 (0.37-1.54)	0.44
Use of Trevira tube				
No†				
Yes	0.7 (0.30-1.59)	0.38	1.6 (0.87-3.05)	0.13

* PJI = periprosthetic joint infection, HR_CS_ = cause-specific hazard ratio, 95% CI = 95% confidence interval, BMI = body mass index, ASA = American Society of Anesthesiologists physical status, and NA = not applicable.

† Reference category.

Early aseptic loosening (Henderson 2A) of the stem occurred in 1 patient (1%), who previously had reconstruction with use of an allograft and total hip replacement that had failed as a result of PJI. After 5 years without further reconstruction, a cemented LUMiC prosthesis was implanted; it was removed 12 months later because of aseptic loosening.

Late aseptic loosening (Henderson 2B) occurred in 4 (2%) of the patients, 3 with an uncemented implant and 1 with a cemented implant. All underwent revision with use of a custom-made implant, 4.4 to 6.4 years after implantation. No complications preceded the aseptic loosening, and none of these patients had undergone reconstruction previously.

Intraprosthetic dissociation (Henderson 3A) occurred in 1 patient (1%), who had persistent subluxation. During revision surgery 32 months after implantation, the LUMiC dual-mobility liner appeared to have dissociated. The stem was well fixed and was left in place, and the liner and cup were revised.

Periprosthetic fracture at implantation (Henderson 3B) occurred in 2 (1%) of the patients; the fractures consisted of a periprosthetic fracture of the ilium around the uncemented stem. One patient underwent successful revision to an uncemented LUMiC stem, implanted slightly more dorsal and lateral in the remaining ilium, utilizing fresh bone stock. One was treated conservatively, but the fracture did not consolidate, resulting in revision to a custom-made prosthesis at 9 months. As a result of dislocation, revision surgery was performed to increase the offset. However, during the procedure, it turned out that the custom-made prosthesis had loosened because of poor bone quality, leading to implant removal and resection arthroplasty.

PJI (Henderson 4) occurred in 41 (25%) of the patients. In 22 (54%) of the patients, PJI occurred within 6 weeks; in 4 (10%), between 6 and 12 weeks; in 2 (5%), between 12 and 24 weeks; and in 13 (32%), at >24 weeks postoperatively. The success rate of ≥1 DAIR procedures was 50% (11 of 22) for patients with an early PJI between 0 and 6 weeks, 75% (3 of 4) with PJI between 6 and 12 weeks, 100% (2 of 2) with PJI between 12 and 24 weeks, and 69% (9 of 13) with PJI at >24 weeks postoperatively. Of the patients with infection following reconstruction, 17 (71%) of 24 without a Trevira tube were successfully managed with DAIR procedures versus 8 (50%) of 16 with a Trevira tube (p = 0.18).

The median duration of the index surgery was 5.5 hours (IQR, 4.3 to 6.5 hours) in patients who developed PJI and 4.8 hours (IQR, 3.6 to 6.5 hours) in those who did not develop PJI (p = 0.13). Surgical duration was not associated with PJI risk (Table IV). The PJI risk was lower for patients with a concomitant proximal femoral reconstruction with silver coating compared with those without silver coating (HR_CS_, 0.2 [95% CI, 0.07 to 0.5]; p < 0.01). Nine (23%) of 40 with silver coating developed PJI compared with 8 (80%) of 10 without silver coating (p < 0.01) (data on silver coating available for 50 of 56 patients with proximal femoral reconstruction). Resections that included the P3 region had an increased PJI risk (HR_CS_, 2.5 [95% CI, 1.4 to 4.7]; p < 0.01). Median blood loss did not differ between patients with PJI (1.9 L [IQR, 1.0 to 2.5 L]) and those without (1.5 L [IQR, 1.0 to 2.3 L]) (p = 0.90). Ultimately, 15 (9%) of the patients underwent revision because of PJI. One had a previous reconstruction (pedestal-cup prosthesis) that had failed because of PJI, and the others did not have previous reconstructions. Four underwent a new reconstruction (3 were revised to a new LUMiC prosthesis during 1-stage [n = 2] or 2-stage [n = 1] revision, and 1 received a custom-made implant). Others underwent resection arthroplasty (n = 8), implant removal and spacer implantation (n = 1), hindquarter amputation (n = 1), or rotationplasty (n = 1) (Table III).

Local recurrence (Henderson 5B) occurred in 13 (8%) of the patients, leading to implant removal in 4 (2%) of the cases (3 hindquarter amputations, 1 resection arthroplasty).

### Cumulative Incidence of Implant Revision, Reconstruction Status, and Functional and Survival Outcomes

**Fig. 1:**
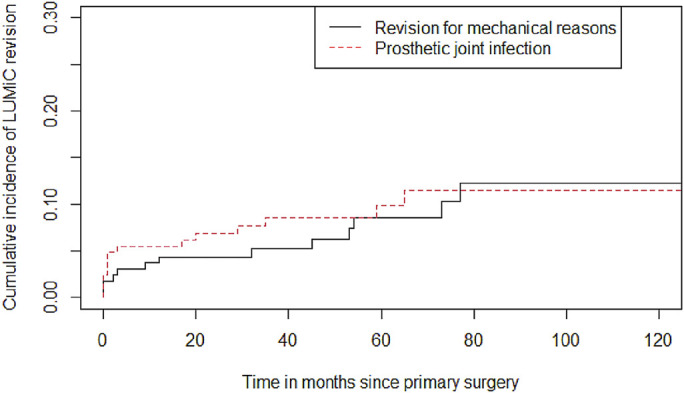
Cumulative incidence of LUMiC revision for mechanical complications (Henderson 1 to 3) and PJI (Henderson 4), using a competing risks model.

The cumulative incidence of implant revision for mechanical reasons (Henderson 1 to 3) at 2, 5, and 10 years was 4% (95% CI, 2% to 8%), 9% (95% CI, 4% to 15%), and 12% (95% CI, 6% to 20%). For PJI (Henderson 4), the rates were 7% (95% CI, 4% to 11%), 10% (95% CI, 5% to 16%), and 11% (95% CI, 6% to 18%) (Fig. 1). For mechanical reasons and PJI (Henderson 1 to 4), the rates were 11% (95% CI, 7% to 17%), 18% (95% CI, 12% to 25%), and 24% (95% CI, 16% to 33%), respectively.

**Fig. 2:**
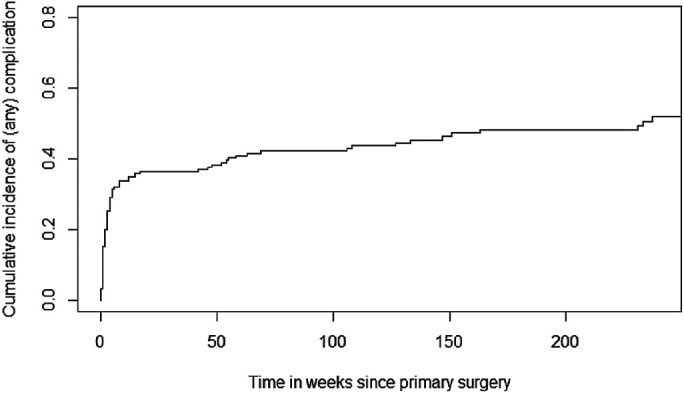
Cumulative incidence of reoperations for any complication, using a competing risks model.

The cumulative incidence of reoperation for any complication at 2, 5, and 10 years was 44% (95% CI, 36% to 51%), 52% (95% CI, 43% to 60%), and 58% (95% CI, 47% to 67%) (Fig. 2).

During the study period, 24 LUMiC reconstructions (14% of the 166 patients) were removed. Four (2%) were removed for tumor progression via hindquarter amputation (n = 3) and resection arthroplasty (n = 1). One additional patient underwent revision for dislocation, but later underwent hindquarter amputation due to tumor progression. Nineteen reconstructions (11%) failed; 11 patients (7%) underwent resection arthroplasty, 5 (3%) were revised to a custom-made prosthesis, 1 (1%) underwent hindquarter amputation, 1 (1%) received a cement spacer, and 1 (1%) underwent rotationplasty (Table III). In 160 (96%) of the patients, limb salvage was achieved. Fifty (30%) were able to walk without mobility aids, 47 (28%) used 1 crutch, 41 (25%) used 2 crutches, and 11 (7%) were not able to walk with crutches (149 with available data).

At the time of the most recent follow-up, 86 (52%) of the patients were alive without disease, 31 (19%) were alive with disease, 41 (25%) had died of disease, and 8 (5%) had died of other causes. The 5-year overall survival was 67% (95% CI, 58.6% to 75.4%).

## Discussion

In the current study, we assessed the mid-term clinical outcomes of patients who underwent reconstruction for periacetabular tumor defects with use of the LUMiC prosthesis. To our knowledge, this is the largest oncological pelvic reconstruction series to date, and we found a substantial reoperation risk but a relatively low revision risk for mechanical complications. Dislocation and PJI remain the major concerns in the early postoperative period.

The dislocation rate (19%) in our cohort is comparable with that found for other stemmed acetabular implants, such as the pedestal-cup and the ice-cream cone prostheses (15% to 26%)^1,4,8,20,21^. Previous surgery at the same site was associated with a higher dislocation risk. This is in line with conventional total hip arthroplasty and might be attributable to the compromised supporting soft tissues^22,23^. We found no association between dislocation risk and the use of a Trevira tube, although use of the Trevira tube might enhance the stability of the construct^12^. The dislocation rate for dual-mobility cups (15%) was substantially higher than the 4% we previously found among 24 dual-mobility cups, which might be attributable to the longer follow-up of the dual-mobility articulations^2^. Although the dislocation risk was not significantly higher for conventional cups (26%), we believe that it is reasonable to continue utilizing the dual-mobility cup. First, previous studies on re-revisions for dislocation in hip-revision surgery have shown promising results for dual-mobility articulations^24-26^. Second, with the exception of a single intraprosthetic dissociation, no cup-specific complications were observed. Caution should be taken when comparing dislocation rates in the literature, since it is unclear if all dislocations (including those managed with closed reduction) are being reported or only those that require revision surgery^21^. Furthermore, most prior studies did not include patients with failed previous reconstructions, while these had a higher dislocation risk in our study (36% versus 11%)^5-7,20,21^. To reduce the dislocation rate, a postoperative abduction orthosis could be of value; Erol et al. found a 10% dislocation risk among 21 patients with LUMiC prostheses, all treated with a hip abduction orthosis for 6 to 12 weeks^7^. Another factor that may contribute to the dislocation risk is the restoration of the center of rotation. Although our study did not assess this aspect, other studies identified it as a risk factor^27,28^. A disadvantage of the LUMiC prosthesis in contrast to custom-made implants is that the vertical shift of the center of rotation is determined by the extent of iliac resection and cannot be adjusted by the length of the stem. A lateral shift of the center of rotation also depends on the cup orientation. On the femoral side, the surgeon may adjust the length and offset of the proximal femoral components to create a more stable situation, although this will not influence the center of rotation.

Stem loosening (Henderson 2) occurred in 3%, comparable with previous results on LUMiC reconstructions (0% to 6%)^2,6,7^ and comparing favorably to those of other techniques, such as the stemmed pedestal-cup prosthesis (6% to 16%) and ice-cream cone prosthesis (8% to 15%)^1,4,8,20,21^. The press-fit fixation of an uncemented hydroxyapatite (HA)-coated stem seems to provide durable fixation.

PJI (Henderson 4A) was the most common complication (25%), leading to implant revision in 9%. Our PJI rate is in line with previously reported results on pelvic reconstructions, which varied between 28% and 33%^2,5,8^. In previous studies, surgical duration was found to be associated with the risk of PJI^23,29^. With the numbers we had, no significant association was identified. Resections including the pubis (P3) had a higher PJI risk. This might be explained by the proximity of the inguinal crease and the creation of a larger wound bed and cavity due to a medial osteotomy. Previous studies did not identify risk factors for PJI, probably because of small sample sizes and the multifactorial etiology. The extent of the resection and the resulting dead spaces, as well as the amount of blood loss, could contribute to development of PJI. Fisher et al. (9%) and Fujiwara et al. (11%) found a relatively low PJI rate in reconstructions with the cemented ice-cream cone prosthesis^1,30^. They believed that this was because of the utilization of antibiotic-laden cement around the cone.

### Limitations

Our study had several limitations. First, the multicenter nature of the study resulted in variations in perioperative treatment protocols, possibly influencing outcomes. Additionally, the inclusion of patients over a 15-year period could present a confounding factor, as there was no accounting for potential secular trends. However, multicenter initiatives over time are needed to obtain sufficient numbers with these lower-incidence oncological conditions, and we present the largest series on pelvic reconstructions to date. Second, the limited number of events per complication did not allow for reliable multivariable analyses. The identification of risk factors remains challenging because of the multifactorial etiology of each complication. Third, there were data lacking concerning the functional outcome scores. Consequently, we added a straightforward question regarding the mobilization status of the patient at the time of the most recent follow-up.

### Conclusions

As with any reconstruction technique for periacetabular tumor defects, we found a substantial reoperation risk. Apart from the reconstruction method used, this seems to be related to the extent of the surgical procedure itself. Nevertheless, efforts should be made to reduce the risk of complications, as these may interfere with the start of adjuvant cancer treatment in some patients. The initial 6 months are critical, as the majority of complications were observed in this period. We found a relatively low risk of mechanical failure in the mid-term, and the majority of patients had their primary implant in situ at the time of the most recent follow-up. On the basis of our findings, patients with previous surgery at the same site have an increased dislocation risk and might benefit from more conservative aftercare. Furthermore, resections involving the P3 region are associated with an increased PJI risk. Future research should focus on the identification of measures to reduce complication rates and enhance implant survival.
